# The Nitrogen Heterocycle Content of Meteorites and Their Significance for the Origin of Life

**DOI:** 10.3390/life8030028

**Published:** 2018-07-11

**Authors:** Zita Martins

**Affiliations:** Centro de Química-Física Molecular-Institute of Nanoscience and Nanotechnology (CQFM-IN) and Institute for Bioengineering and Biosciences (iBB), Departamento de Engenharia Química, Instituto Superior Técnico (IST), Universidade de Lisboa, 1049-001 Lisboa, Portugal; zita.martins@tecnico.ulisboa.pt

**Keywords:** nitrogen heterocycles, meteorites, life, prebiotic chemistry

## Abstract

Carbonaceous chondrites are very primitive meteorites that are rich in carbon. They contain many soluble organic compounds, including nitrogen heterocycles. These play a crucial role in present-day living organisms as they are components of the genetic material and of the co-factors of enzymes. This review outlines the nitrogen heterocycle content of carbonaceous meteorites. The potential mechanisms of formation of these molecules are also described. Measurements of the compound-specific carbon and hydrogen isotopic compositions are mentioned as a way of establishing the origin of the nitrogen heterocycles detected in meteorites.

## 1. Introduction

Meteorites are extraterrestrial objects originating from comets or asteroids that survive the passage through the Earth’s atmosphere and impact the Earth’s surface. They are named after the town or geographic feature in which they are found. The names of Antarctic meteorites are followed by a number, in which the first two digits correspond to the year the meteorite was found, and the last three digits correspond to the specimen number. Around 86% of all meteorites that fall to the Earth are chondrites. Their parent bodies have not experienced melting, and they are named after the millimeter-sized spherules (i.e., chondrules) that they contain. The parent bodies of the remaining 14% of meteorites that fall to the Earth have experienced melting and differentiation [1]. Chondrites can be divided into ordinary (O), enstatite (E), carbonaceous (C), Rumuruti (R), and Kakangari (K) chondrites, with the ordinary, enstatite, and carbonaceous chondrites further divided into different groups according to their mineralogy and bulk chemical composition [1,2,3]. Carbonaceous chondrites are the most primitive meteorites because their bulk chemical compositions match that of the solar photosphere (except for the gaseous elements, e.g., H, He, etc.) more closely than any other class of chondrites [4]. The division of the carbonaceous chondrites into groups include the ones named after its type specimen (CI, CM, CK, CO, CR, CV), some unusual ones that have been affected by impact processes (CH and CB), and several ungrouped members [5]. They are further grouped into petrographic types (ranging from 1 to 6) depending on the intensity of thermal metamorphism or aqueous alteration on their parent bodies. Thermal metamorphism is the adjustment of the minerals due to the increased temperatures in the meteorite parent body, while aqueous alteration is the modification of the original minerals into a new assemblage of minerals, due to the reaction with water at low temperature in the meteorite parent body. A petrologic type from 3 to 1 indicates increasing aqueous alteration, while a petrologic type from 3 to 6 indicates increasing thermal metamorphism [6,7]. These processes may influence the chemical compositions of carbonaceous chondrites [7,8,9]. Carbonaceous chondrites have high carbon content (~3.5 wt. %) [10], which may be present in different forms, including organic matter [11,12,13]. More than 70% of this organic matter is in the solvent-insoluble form [14,15,16], while the remaining 30% is composed of several solvent-soluble organic compounds [17,18,19].

Nitrogen heterocycles, i.e., cyclic compounds that have at least one nitrogen atom along with carbon atoms as members of their rings, have been detected in the solvent-soluble organic fraction of carbonaceous chondrites. The nitrogen heterocycles detected in carbonaceous meteorites include pyridine carboxylic acids, diketopiperazine, hydantoins, purines, pyrimidines, triazines, pyridines, quinolines, carboxylactams, lactams, lactims, and the amino acid proline. Some of these compounds may have been directly involved in primitive biological systems, as in present-day nitrogen heterocycles are part of the co-factors of several enzymes as well as of genetic material (Ribonucleic acid (RNA) and Deoxyribonucleic acid (DNA)) [20]. The exogenous delivery of these meteoritic molecules between 4.56 to 3.8 billion years ago may have contributed to the inventory of compounds from which life may have emerged between 3.8 to 3.5 billion years ago [21,22,23,24,25,26,27,28,29,30,31,32]. Therefore, it is important to determine which nitrogen heterocycles are present in carbonaceous meteorites, which ones are indigenous, and what were their formation mechanisms. Measurement of the compound-specific carbon, hydrogen, or nitrogen isotopic compositions are usually used to determine if organic compounds detected in carbonaceous chondrites are indigenous or terrestrial contamination.

$$\delta \left(‰\right)=\frac{\left(R_{sample}-R_{standard}\right)}{R_{standard}}\times 1000$$

The stable isotope compositions are given in δ values (‰), where *R* represents D/^1^H for hydrogen, ^13^C/^12^C for carbon, or ^15^N/^14^N for nitrogen. The following standards are used: Vienna standard mean ocean water (VSMOW) for hydrogen, Vienna Pee Dee Belemnite (VPDB) for carbon, and atmospheric nitrogen for nitrogen. Indigenous organic compounds are enriched in D, ^13^C, and ^15^N (e.g., [17,33,34,35]).

Meteoritic soluble organic compounds are thought to be formed by different processes: low temperature reactions in the interstellar medium (supported by D and ^15^N enrichments [36,37,38,39,40,41]) and subsequent meteorite parent body accretion, aqueous alteration in the meteorite parent (i.e., melting of ice in the asteroid due to heating from the decay of several short-lived radionuclides, such as ^26^Al [42]), or a combination of these two mechanisms [17,41]. This manuscript reviews the nitrogen heterocycle content of carbonaceous meteorites and their formation mechanisms.

## 2. Inventory of Meteoritic *N*-Heterocycles 

### 2.1. Pyridine Carboxylic Acids

**Figure 1:**
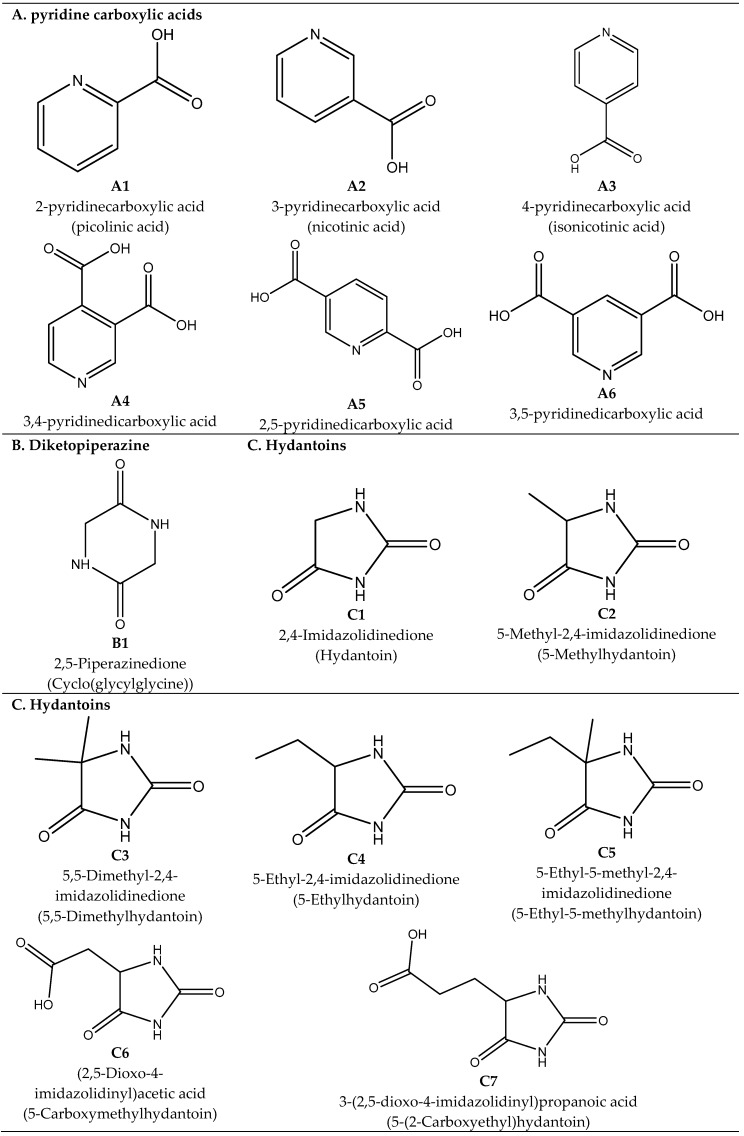

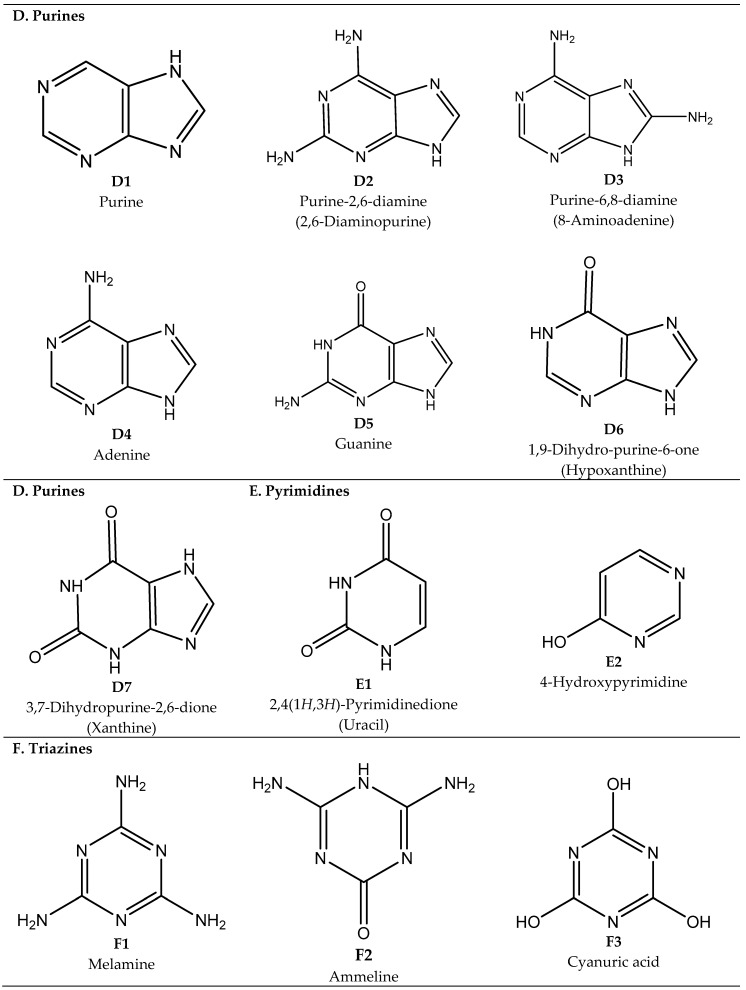

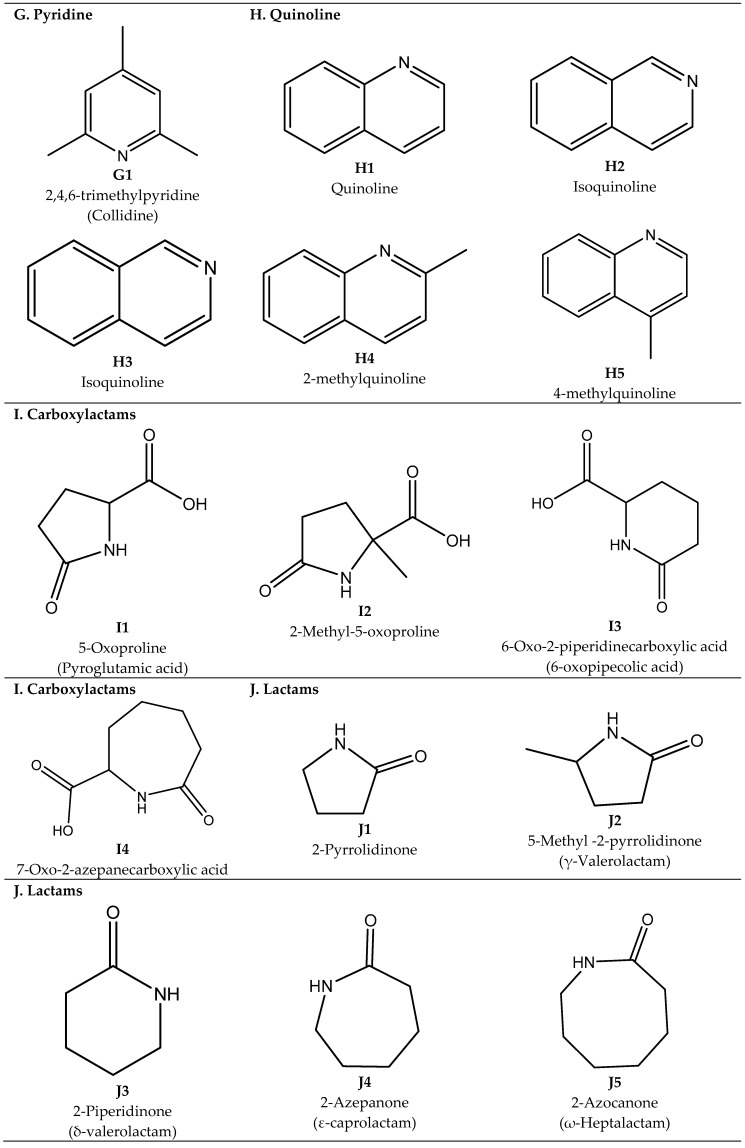

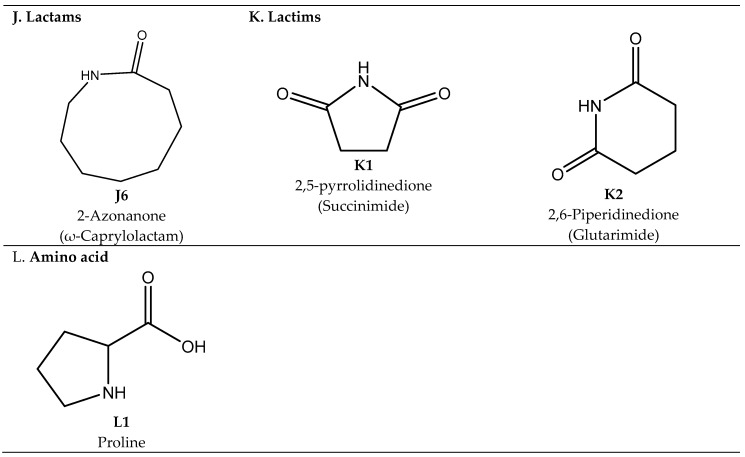
The structure and the International Union of Pure and Applied Chemistry (IUPAC) name (and other commonly known name) of the nitrogen heterocycles detected in carbonaceous chondrites. Compounds include pyridine carboxylic acids (**A1** to **A6**), a diketopiperazine (**B1**), hydantoins (**C1** to **C7**), purines (**D1** to **D7**), pyrimidines (**E1** and **E2**), triazines (**F1** to **F3**), pyridine (**G1**), quinolines (**H1** to **H3**), carboxylactams (**I1** to **I4**), lactams (**J1** to **J6**), lactims (**K1** to **K2**), and amino acid proline (**L1**).

Pyridine monocarboxylic acids have been detected in several carbonaceous chondrites [43,44,45,46]. They were first detected in Tagish Lake (C2-ungrouped) and Murchison (CM2), with total abundances of 7.5 and >7 parts-per-million (ppm), respectively [43]. Individual pyridine monocarboxylic acids present in the water extracts of these two meteorites included nicotinic acid (3-pyridinecarboxylic acid) and its two isomers (2-pyridinecarboxylic acid and 4-pyridinecarboxylic acid (Figure 1, structures **A1**–**A3**), and at least 12 methyl- and dimethyl-homologs [17]. Some of these compounds were found to be extra-terrestrial, as shown by the high positive values of δ^13^C = +20.3‰ ± 1.7 and δD = +129 ± 1‰ measured for nicotinic acid in Murchison [44,45]. A value of δ^13^C = +20.3 ± 1.2‰ was determined for one of the methyl homologues of nicotinic acid in Murchison (CM2), and a value of δD = +621 ± 43‰ was measured in Murray (CM2) (Table 1) [44,45]. Eight Antarctic CM2 meteorites were further analysed for pyridine monocarboxylic acids: Allan Hills (ALH) 85013, Dominion Range (DOM) 03183, DOM 08003, Elephant Moraine (EET) 96016, LaPaz Ice Field (LAP) 02333, LAP 02336, Lewis Cliff (LEW) 85311, and Wisconsin Range (WIS) 91600. The three structural isomers were present in formic acid extracts of all these meteorites (Table 2) [46]. In addition, pyridine dicarboxylic acids (3,4-pyridinedicarboxylic acid, 2,5-pyridinedicarboxylic acid, and 3,5-pyridinedicarboxylic acid) were unambiguously identified in multiple of those meteorite extracts [46] (Figure 1, structures **A4**–**A6**).

#### Synthesis of Pyridine Carboxylic Acids

Aqueous alteration in the meteorite parent body seems to influence the synthesis of pyridine carboxylic acids, with a decrease of their abundances with increasing aqueous alteration, i.e., aqueous alteration may have had a destructive effect on these compounds [46]. In addition, it was proposed that the oxidation of alkylpyridines would give pyridine carboxylic acids [47]. Although to date no pyridine carboxylic acids or their potential precursors (e.g., pyridine) have been detected in the interstellar medium, they may have been formed by radiation of icy interstellar grains. To test this, a 1:1 ice mixture of pyridine and CO_2_ was proton-irradiated [46]. Pyridine monocarboxylic acids were later identified in the corresponding non-volatile residue, with distributions similar to the ones detected in carbonaceous meteorites, which suggested that interstellar chemistry may have contributed to the formation of meteoritic pyridine carboxylic acids. However, it was pointed out that more realistic interstellar ice compositions should be tested before drawing a conclusive remark [46].

### 2.2. Diketopiperazine and Hydantoins

2,5-Piperazinedione (cyclo(Gly-Gly)) was the only diketopiperazine detected in carbonaceous chondrites (Figure 1, structure **B1**). It was present in the Yamato-791198 (CM2) and Murchison (CM2) meteorites at the concentrations of 2.1 and 2.6 parts-per-billion (ppb), respectively (Table 3) [48]. Hydantoins were also detected in the same meteorite extracts, with concentrations ranging from 1.0 to 6.5 ppb in Yamato-791198, and from 0.9 to 11.9 ppb in Murchison (Table 3) [48]. Detected compounds included hydantoin, 5-methylhydantoin, 5,5-dimethylhydantoin, 5-ethylhydantoin, 5-ethyl-5-methylhydantoin, 5-carboxymethylhydantoin, and 5-(2-carboxyethyl)hydantoin (Figure 1, structures **C1**–**C7**). Some of these were previously detected in Murchison, but their concentration was not determined [49].

#### Synthesis of Diketopiperazine and Hydantoins

The only detected diketopiperazine (2,5-piperazinedione) in carbonaceous meteorites may have been formed by oligomerization of amino acids via drying-wetting cycles in the presence of inorganic materials (e.g., clay minerals) [50,51,52,53]. Glycine is more reactive than other amino acids to form dipeptides by oligomerization on the surface of a clay mineral, which could explain why 2,5-piperazinedione (cyclo(Gly-Gly)) was the only detected diketopiperazine in meteorites [48,51]. Hydantoins, which were present in the same extract as diketopiperazine, were suggested to be formed by intramolecular dehydration of *N*-carbamyl amino acids in the parent body of Murchison [49]. However, analysis by Shimoyama and Ogasawara [48] did not support formation of hydantoin through *N*-carboxyanhydrides of glycine. Indeed, while the concentrations of hydantoin and 5-methylhydantoin were roughly equal in the Yamato-791198 meteorite (Table 3), only Gly-Gly was detected (no glycylalanine, alanylglycine, and alanylalanine were detected) [48]. Alternatively, vacuum UV photo-irradiation of interstellar/circumstellar ice analogues containing H_2_O, CH_3_OH and NH_3_ has been shown to form small quantities of hydantoin [54].

### 2.3. Purines, Pyrimidines, Triazines, Pyridines, and Quinolines

Several researchers have detected purines, pyrimidines, triazines, pyridines, and quinolines in carbonaceous chondrites (Figure 1, structures **D1** to **H3**) [47,55,56,57,58,59,60,61,62,63,64,65,66]. Throughout the years, there has been controversy regarding the detection of these compounds, as different research groups found different purines, pyrimidines, and triazines, in some cases for the same meteorite. Purines (adenine and guanine) and triazines (melamine and ammeline) (Figure 1, structures **D4**, **D5**, **F1**, **F2**, respectively) were detected in the Orgueil meteorite (CI1) by Hayatsu and co-authors [55,56]. Adenine, guanine, melamine, and cyanuric acid (Figure 1, structure **F3**) were also detected in Orgueil by the same research group when using drastic extraction conditions (hot temperature, 3–6 M HCl or CF_3_COOH) [57]. On the other hand, Folsome and co-authors found 4-hydroxypyrimidine (Figure 1, structure **E2**) and heterogenous classes of speculative pyrimidines in Murchison (CM2), Murray (CM2), and Orgueil, which did not agree with the results of Hayatsu and co-authors [58,59,60]. The explanation for this discrepancy was found a few years later. Xanthine, and tentatively guanine and hypoxanthine (Figure 1, structures **D7**, **D5**, **D6**, respectively) were detected in formic acid extracts of the Murchison meteorite by dual-column, ion-exclusion chromatography and ultraviolet spectroscopy [61]. Hydroxypyrimidines were detected only after the silylation of a water extract, suggesting that the compounds previously detected were terrestrial contaminants from the silylation reagent [61]. Uracil (Figure 1, structure **E1**) was detected for the first time in water and formic acid extracts of Murchison, Murray, and Orgueil using fractionation techniques and ion exclusion chromatography with UV spectroscopy [62]. Orgueil contained 27 ppb, while Murchison contained 33 ppb of uracil (Table 4). Adenine, guanine, hypoxanthine, and xanthine were also detected in formic acid extracts of these three meteorites [63]. Triazines were not detected and, similarly to hydroxypyrimidines, were suggested to be terrestrial contamination resulting from the experimental procedures used previously [55,56]. The efficiency of the extraction procedure for purines, pyrimidines and triazines was determined by extracting a sample of the Allende meteorite (CV3), which was spiked with known amounts of standard compounds [63]. Recoveries of these compounds for the water extraction after desalting ranged from 41% to 81%, while for formic acid, it had an average recovery of 71% [63]. A conclusion about the origin of purines and pyrimidines detected in meteorites was obtained in 2008 when compound-specific carbon isotope measurements of these compounds were performed by using gas chromatography-combustion-isotope ratio mass spectrometry (GC-C-IRMS) [65]. Carbon isotope ratios of uracil and xanthine in Murchison (δ^13^C = +44.5‰ and +37.7‰, respectively), and of uracil and thymine from a soil collected in the proximity of the meteorite fall site (δ^13^C = −10.6‰ and −15.9‰, respectively) showed that uracil and xanthine detected in the Murchison meteorite were enriched in ^13^C and therefore were indigenous to this meteorite (Table 5) [65]. Purines and pyrimidines were detected in several other meteorites. Guanine, and possible xanthine and hypoxanthine, were detected in formic acid extracts of Y-74662 (CM2) and Y-791198 (CM2) [64]. Purine, purine-2,6-diamine, purine-6,8-diamine (Figure 1, structures **D1** to **D3**), adenine, guanine, hypoxanthine, and xanthine were detected in some of the following meteorites: Orgueil (CI1), Meteorite Hills (MET) 01070 (CM1), Scott Glacier (SCO) 06043, (CM1) Allan Hills (ALH) 83100 (CM1/2), Lewis Cliff (LEW) 90500 (CM2), Lonewolf Nunataks (LON) 94102 (CM2), Murchison (CM2), Grosvenor Mountains (GRO) 95577 (CR1), Elephant Moraine (EET) 92042 (CR2), Graves Nunataks (GRA) 95229 (CR2), and Queen Alexandra Range (QUE) 99177 (CR2) [66]. The total purine abundances were up to 12 times higher in CM2 carbonaceous chondrites than in the other analysed meteorites (Table 4, [66]). Within CM chondrites, the abundances and diversity of purines decreased with increasing aqueous alteration [66]. Pyridines (2,4,6-trimethylpyridine) (Figure 1, structure **G1**) and quinolines (quinoline, isoquinoline, 2-methylquinoline and 4-methylquinoline) (Figure 1, structures **H1** to **H5**) were also detected in Murchison [47].

#### Synthesis of Purines, Pyrimidines, Pyridines, and Quinolines

Only upper limits of pyrimidines, pyridine, quinoline, and isoquinolines were detected in the gas phase of astrophysical environments [67,68,69,70], which may be explained by their low stability against UV radiation in these environments [71,72]. Laboratory work has demonstrated that uracil, cytosine, and thymine may be formed by UV photoirradiation of astrophysical ice analogues containing pyrimidine [73,74,75,76]. UV photoirradiation of purine mixed with combinations of H_2_O and NH_3_ ices resulted in the formation of adenine, guanine, and other purine derivatives [77]. The formation of adenine, guanine, and analogues in a UV-irradiated mixed H_2_O:NH_3_ ice (10:1) containing purine was confirmed by using *ab initio* and density functional theory computations [78]. These investigations suggested a multistep reaction mechanism involving water, ammonia, a purine cation, and hydroxyl and amino radicals. They predicted that mono-substituted products (preferentially adenine and 2-hydroxypurine) were more energetically favourable, followed by bi-substituted product (preferentially isoguanine and xanthine) [78]. Experimental results were in agreement with *ab initio*, with adenine and hypoxanthine as the most abundant products, followed by the bi-substituted photoproducts [77]. Purines and pyrimidines may also be formed by polymerization of ammonium cyanide solutions at temperatures ranging from −78 °C to +80 °C, which could have happened in the parent body of carbonaceous chondrites [79,80,81,82,83,84,85,86,87,88,89]. Thermochemical computational simulations show that Fischer-Tropsch type (FTT) synthesis is likely the dominant source of nucleobases within a meteorite parent body (planetesimal) model, followed by non-catalytic synthesis (under certain chemical conditions) [90]. Pyridine, quinoline, and isoquinoline can be formed from the UV irradiation of benzene and naphthalene in H_2_O and H_2_O:NH_3_ ices at low temperature [91]. This study also showed that these compounds may be formed in icy grains without requiring that they be formed in or condense from the gas phase, therefore avoiding photodegradation [91]. Other suggested formation mechanisms of pyridines include FTT reactions, e.g., the synthesis of alkyl pyridines by catalytic reactions of aldehydes and ammonia [47].

### 2.4. Carboxylactams, Lactams, and Lactims

Several carboxylactams were detected in the Murchison meteorites, including 5-oxoproline, 2-methyl-5-oxoproline, 6-oxo-2-piperidinecarboxylic acid, and 7-oxo-2-azepanecarboxylic acid (Figure 1, structures **I1** to **I4**) [49]. Although no abundances were determined for this class of compounds, results suggest that the alkyl-substituted five-and six-membered ring carboxy lactams are the most abundant. Lactams are also present in the Murchison meteorite, including the five-membered ring compound 2-pyrrolidone to at least the nine membered ring compound 2-azonanone (Figure 1, structures **J1** to **J6**). Both lactams and carboxylactams present in Murchison include most of their structural isomers based on mass spectra and retention time comparison to those of silylated standard compounds or mass spectra with the expected fragment ions [49]. For the carboxylactams, the seven-carbon isomers (i.e., alkyl-substituted five-and six-membered ring carboxy lactams) are the most abundant members of the series, and a nine-carbon homologue is the highest member of the series. The lactams start with the five-membered ring compound and extends to at least the nine membered ring compound (8-octanelactam) [49]. Finally, 2,5-pyrrolidinedione and 2,6-piperidinedione (two lactims) were also positively identified in the Murchison meteorite (Figure 1, structures **K1** and **K2**).

#### Synthesis of Carboxylactams, Lactams, and Lactims

The mechanisms for the synthesis of carboxylactams, lactams and lactims in carbonaceous meteorites have been proposed [49]. Carboxylactams were suggested to be formed by dehydration of *N*-carbamyl amino acids (formed by the reaction of cyanates with amino acids), with subsequent decarboxylation leading to lactams. Alternatively, carboxylactams would be formed via dehydration of the corresponding amino acid. The reaction of hydantoins with cyanates would form an acid amide derivative, which would subsequently synthesize a lactim via dehydration [49].

### 2.5. Amino acid Proline

The amino acid proline (Figure 1, structure **L1**) was detected in Murchison and Murray [92,93]. Concentrations of proline ranged from 622 to 1550 ppb in the Murchison meteorite, while it was 400 ppb in Murray [93,94,95,96]. The indigenous nature of proline in Murchison was indicated by its stable nitrogen isotope composition (δ^15^N value of +50‰ for d,l-proline) [97].

#### Synthesis of the Amino Acid Proline

Amino acids may be formed by UV photolysis of interstellar ice analogs. In fact, proline was found in the residues of interstellar ice analogs processed with UV radiation [98,99]. A radical-radical mechanism of formation of amino acids in interstellar ice analogs was proposed [100]. It was later shown that a modified radical-radical mechanism could happen, but general amino acid formation occurs via multiple pathways [101]. A more recent study using chemical retrosynthesis shows that proline in interstellar ices may be formed from the amine precursor butylamine [102].

## 3. Astrophysical and Astrobiological Implications of Meteoritic *N*-Heterocycles

Several *N*-heterocycles have been detected in carbonaceous chondrites, and their synthesis may have an interstellar heritage via low temperature reactions, followed by accretion to the meteorite parent body. Furthermore, aqueous alteration in the meteorite parent may have also played a role on its own on the synthesis of *N*-heterocycles or after interstellar medium synthesis and accretion [17,41]. Although compound-specific carbon, hydrogen, or nitrogen isotopic compositions have been performed for *N*-heterocycles, the available data are extremely limited and were obtained for only a handful of individual compounds [44,45,65,96] (Table 1 and Table 5). The lack of an extensive study on the simultaneous analysis of at least two sets of data of the compound-specific carbon, hydrogen, or nitrogen isotopic compositions for all the detected classes of meteoritic *N*-heterocycles forfeits a complete understanding of the mechanisms of formation of these compounds. This research is certainly worth exploring in the future to fully master their formation mechanisms.

The prebiotic contribution of meteoritic *N*-heterocycles must be viewed first in light of their solubility on the oceans of the primitive Earth, i.e., how easily they are extracted from carbonaceous chondrites and could be used in chemical reactions on our early planet, followed by their potential use by primitive living organisms. Some *N*-heterocycles (e.g., pyridine carboxylic acids, hydantoins, purines, pyrimidine, etc.) are soluble in water, which means that they could potentially participate in primitive biological systems. However, the abundances of individual *N*-heterocycles in carbonaceous chondrites are low, in the order of a few ppb (Table 2, Table 3 and Table 4), which indicates that carbonaceous meteorites were probably not the only source of *N*-heterocyles on the early Earth. In addition, it has not yet been found how to synthesise ribonucleotides from nucleobases (and ribose) under prebiotic conditions, as the addition of nucleobases to ribose is either inefficient or does not occur [103,104]. Nevertheless, none of these preclude the use of meteoritic *N*-heterocycles on primitive biological systems. Indeed, in present-day biology, the amino acid proline is used in the biosynthesis of proteins, while *N*-heterocycles are components of the co-factors of enzymes and of genetic material (RNA and DNA) [20].

## 4. Conclusions

This manuscript reviews the nitrogen heterocycle content of meteorites, including the abundances and the compound-specific carbon and hydrogen isotopic compositions of individual compounds in the soluble organic fraction. Likely indigenous meteoritic nitrogen heterocycles included pyridine carboxylic acids, piperazinedione, hydantoins, purines, pyrimidines, pyridines, quinolines, carboxylactams, lactams, lactims, and proline. Formation mechanisms included reactions in the interstellar medium, followed by accretion and processing on the meteorite parent body. While likely not the only source of *N*-heterocycles used by primitive living organisms, carbonaceous meteorites contributed to the feedstock of organic molecules freely available on the early Earth.

## Figures and Tables

**Table 1:** Compound specific stable isotope composition (‰) of individual pyridine carboxylic acids in Murchison and Murray [44,45].

	Murchison		Murray	
	δD	δ^13^C	δD	δ^13^C
Nicotinic acid	+129 ± 1	+20.3 ± 1.7	-	-
Nicotinic methyl homologue	-	+20.3 ± 1.2	+621 ± 43	-

**Table 2:** The abundances (ppb) of pyridine carboxylic acids in the formic acid extracts of carbonaceous meteorites [46].

	WIS 91600	DOM 03183	DOM 08003	ALH 85013	EET 96016	LAP 02333	LAP 02336	LEW 85311
Picolinic Acid	25.1 ± 2	70.2 ± 7	482.2 ± 48	98.8 ± 10	322.0 ± 32	197.1 ± 20	318.4 ± 32	510.7 ± 51
Nicotinic Acid	96.3 ± 10	121.9 ± 12	221.0 ± 22	139.6 ± 14	265.1 ± 26	246.8 ± 25	332.1 ± 33	571.8 ± 57
Isonicotinic Acid	42.0 ± 42	70.8 ± 7	153.7 ± 15	67.4 ± 7	116.7 ± 12	161.5 ± 16	256.9 ± 26	294.1 ± 29

**Table 3:** Concentration (ppb) of a piperazine and the hydantoins in Yamato-791198 and Murchison [48].

	Yamato-791198	Murchison
Cyclo(glycylglycine)	2.1	2.6
Hydantoin	6.5	7.3
5-Methylhydantoin	5.5	11.9
5,5-Dimethylhydantoin	5.6	9.0
5-Ethylhydantoin	1.0	1.5
5-Ethyl-5-methylhydantoin	3.4	6.7
5-Carboxymethylhydantoin	n.d.	0.9
5-(2-Carboxyethyl)hydantoin	n.d.	1.4

n.d.—Not detected above 0.9 ppb.

**Table 4:** The abundances (ppb) of purines [66] and a pyrimidine [62] in formic acid extracts of carbonaceous meteorites. Numbers in parentheses represent concentrations for tentative structural assignments (i.e., they were usually due to low S/N coupled with a complex multiple reaction monitoring (MRM) chromatogram, not allowing for unambiguous assignment). The + sign indicates the positive identification for the compound (but no quantitation).

	Orgueil	SCO 06043	MET 01070	GRO 95577	ALH 83100	Murchison	LEW 90500	LON 94102	GRA 95229	EET 92042	QUE 99177
Guanine	20	(2)	29	<2 ^†^	21	56	167	244	4	<2 ^†^	<2 ^†^
Hypoxanthine	(5)	(4)	<3 ^†^	<3 ^†^	4	26	23	94	(4)	<3	<3 ^†^
Xanthine	<10 ^†^	<10 ^†^	<10 ^†^	<10 ^†^	(4)	60	22	77	<10 ^†^	<10 ^†^	<10 ^†^
Adenine	7	4	5	<0.5	1	5	10	30	21	5	11
Purine	5	<1 ^†^	<1 ^†^	<1 ^†^	<0.1 ^†^	3	1	6	9	(4)	7
2,6-Diamonopurine	<2 ^†^	<2 ^†^	<2 ^†^	<2	<0.2 ^†^	+	<0.2 ^†^	5	<2 ^†^	<2 ^†^	<2 ^†^
Uracil	27	n.d.	n.d.	n.d.	n.d	33	n.d.	n.d.	n.d.	n.d.	n.d.

^†^—Not detected and reported as upper limit. n.d.—not determined.

**Table 5:** Compound-specific stable isotope composition (δ^13^C in ‰) of uracil and xanthine in Murchison [65].

	Murchison
**Uracil**	+44.5 ± 2.3
**Xanthine**	+37.7 ± 1.6
